# Over the Hills and Through the Hollers: How One Program is Assisting Residents of Appalachian with Opioid Use Recovery

**DOI:** 10.13023/jah.0403.05

**Published:** 2023-01-01

**Authors:** Aubrey E. Jones, Jayme E. Walters, Aaron R. Brown

**Affiliations:** University of Kentucky, aubrey.jones12@uky.edu; Utah State University; Western Carolina University

**Keywords:** Appalachia, Rural Social Work, Substance Use, Opioid Use Disorder

## Abstract

**Introduction:**

The consequences of increasing opioid misuse in the U.S. over the last two decades have been severe, contributing to hundreds of thousands of lives lost and heavy tolls on individuals, families, and society. The Appalachian Region has been hit particularly hard, with its predominantly rural landscape seeing disproportionate increases in opioid misuse and overdoses. These cases have been difficult to address due to poor treatment access and capacity constraints in many areas of Appalachia.

**Purpose:**

The current study focuses on evaluating The Kentucky Access to Recovery Program (KATR), which provides services to individuals recovering from opioid addiction residing in several counties in Eastern Kentucky. Its purpose is to understand the impact of KATR on service recipients’ access to recovery services and supports.

**Methods:**

Semi-structured interviews were conducted with 12 service recipients, three service providers, and four vendors of support services related to housing, transportation, medical/dental care, employment, and childcare. Qualitative data were analyzed using thematic analysis.

**Results:**

Themes related to individual-level impacts were identified and discussed, including behavioral changes related to recovery, physical and mental health improvements, relationship repair, regaining custody of children, provision of needed supports, and ability to gain employment and improve finances. Study findings showed that KATR had meaningful impacts on the lives of service recipients by helping meet needs and reducing barriers to their ongoing recovery.

**Implications:**

Through its use of vouchers for support services and basic-needs provision, KATR demonstrates a potentially effective strategy for increasing access to health-related social services for persons in recovery in predominantly rural areas.

## INTRODUCTION

The misuse of prescription and illicit opioids has become a nationwide epidemic. Since 1999, opioid-related overdose deaths have more than quadrupled.^1^ Specifically, opioid-related deaths have risen from 8,050 deaths in 1999 to 68,630 recorded opioid-related deaths in 2020.^1^ When the opioid overdose crisis began in the early part of the 21^st^ century, most overdoses were related to the misuse of prescription opioids; however, as the crisis has progressed, overdoses have increasingly involved heroin, and more recently, illicitly manufactured fentanyl.^2^ Current estimates indicate that 9.5 million Americans over the age of twelve misused opioids in the past year.^3^

Consequences of opioid misuse can be life-threatening, as this behavior coincides with a significant rise in both morbidity and mortality, exacting a heavy toll on patients, physicians, and societal efficiency. Other societal costs that exist due to the opioid epidemic include declines in work productivity and attendance, as well as increases in use of emergency services, correctional facilities, and public insurance.^4^–^5^ Increasingly, children of parents with challenges related to opioid misuse are finding themselves in the child welfare system.^6^ As opioid misuse continues to rise, many children are at risk of developing behavioral disorders related to the exposure to prenatal substances and access of household substances. For all these reasons, mediation of the opioid epidemic is imperative to child welfare, stability of families, and societal order.

### Appalachia and the Opioid Crisis

The Appalachian Region is highly rural, with 42% of its area classified as rural compared to 20% of the U.S. as a whole.^7^ The region’s unique geography and characteristics present challenges that contribute to the opioid epidemic, such as substantial decline of the regional economy (e.g., the closure of mines and manufacturers), extreme and persistent poverty, generational addiction, lack of access to healthcare services and preventive programs, and limited recreation and entertainment opportunities.^7^–^8^ The Appalachian Region has some of the highest opioid prescribing rates in the country, and these contribute to high levels of misuse and mortality due to drug overdoses—37% higher when compared to the U.S. as a whole.^7^–^10^

The present study considers how this crisis unfolds in Eastern Kentucky, the 31-county Appalachian portion of one U.S. state. It examines an agency which provides services to one Eastern Kentucky county and the four nearest surrounding counties. These five counties have experienced substantial effects from the opioid crisis across the past decade. In County 1, the age-adjusted rate of drug overdose deaths in 2018 was approximately 60% higher than the national average.^9^ As the economy has diminished, so has the health and opportunities of residents in County 1, which now ranks in the top 50 in the nation for risk of HIV or Hepatitis C outbreaks as a result of opioid misuse.^10^ County 2, which neighbors County 1, has experienced a dramatic increase in unemployment, and this has subsequently instilled a “sense of hopelessness” amongst the citizens.^11^ The unemployment crisis has only made the opioid and drug problem worse.^11^ County 2 has also experienced a rise in Hepatitis C, a consequence of shared needles for drug use. From 2015 to 2019, all five counties had poverty rates that were much higher than for the U.S. as a whole, averaging 125% higher than the national average poverty rate of 13.4%.^12^ As of 2020, the Appalachian Regional Commission has designated all five counties as “economically distressed,” meaning they rank in the worst 10 percent of the nation’s counties.^13^

### The Kentucky Access to Recovery (KATR) Program

The Kentucky Access to Recovery (KATR) program helps men and women recovering from opioid addiction. All services offered are free to low-income adult residents of Kentucky who are in treatment or early recovery (2 years or less). KATR has three offices throughout Eastern Kentucky. The offices offer funds for various services: the provision of clothing, support locating housing, medical/dental care, transportation assistance, vocational/employment services, and childcare assistance. These services are offered to eligible participants who are resident in counties in and adjacent to KATR offices. The resources and material support must be connected to the eligible participant’s treatment and/or recovery management plan. KATR has been established as a supplement and not a replacement plan, meaning other resources—such as insurance, Medicaid, Medicare, and state block grant dollars—must be used first.

The authors conducted a program evaluation of KATR. The evaluation considered program effectiveness in its entirety, the effects on the program related to the social determinants of health, and how the program impacts individuals. The purpose of this study is to understand the impact of KATR on service recipients and the barriers they encounter in their recovery journey while part of the program.

## METHODS

### Sample and Sample Recruitment

To achieve the study goals, semi-structured interviews were conducted with three primary groups of individuals involved with the KATR program: (1) 12 service recipients—persons who received services from KATR; (2) three service providers—the program coordinators of KATR; and (3) four vendors of support services related to mechanical needs, housing assistance, dental work, and childcare. Service recipients were randomly selected to participate in the study. Using a random number generator, fifty participants were identified to receive the initial recruitment postcard and a follow-up phone call. Interviews were scheduled or conducted on the phone during the first initial phone contact which took place approximately three to five days after postcards were received. For program coordinators and vendors, investigators were provided contact information, and all potential respondents were emailed about the current study.

### Data Collection

Semi-structured interviews were completed with all three groups from August to October 2020 via telephone or Zoom and recorded via Zoom. When recording the telephone calls, the interviewer opened Zoom and used Zoom to record while conducting the interview on speaker phone in a secure and private office space. Participants were given the option to decline the recording of their interview. All participants provided consent to have their interview recorded. Interviews with program coordinators lasted about an hour, and vendor interviews lasted approximately 15 minutes. The service participant interviews averaged about 15 minutes in length. The Zoom recordings were saved as MP3 files without video. Recordings were transcribed primarily using Rev, a third-party transcribing service, and reviewed by the research team for accuracy. Each participant group addressed topics related to the impact of the opioid support services program on the service users, community, and organization (see Additional Files for interview guides).

#### Service Recipients

Interviews with service recipients lasted, on average, 15 to 20 minutes. Service recipients received a $15 gift card to Walmart for participating in the study. Three of the authors conducted the semi-structured interviews with service recipients until data saturation was reached. The three authors discussed weekly the context of the interviews until all authors agreed that data saturation had been reached for this subset of respondents.

#### Program Coordinators

All interviews with the program coordinators were conducted by one of the investigators and lasted approximately 50 minutes each. No incentives were provided to program coordinators.

#### Vendors

One of the authors conducted semi-structured interviews with four of the KATR participating vendors. Each interview lasted, on average, 15 minutes. Incentives were not provided for vendors.

### Data Analysis

Data analysis was guided by the social determinants of health (SDOH) framework which includes five domains: economic stability; education access and quality; healthcare access and quality; neighborhood and built environment; and social and community context.^14^ A two-cycle coding approach was applied to surface key themes. Descriptive coding was employed in the first round of coding; in this process, one-word (or short) phrases were assigned to data. Pattern coding was then applied by the investigators sifting and sorting the first-cycle codes into patterns.^15^ Two investigators separately coded the data. After the first round of coding, they met to discuss codes and address discrepancies. Similarly, after the first round, two investigators reduced the codes into patterns and met to assess consistency of their patterns. Finally, thematic analysis was used to determine common themes from the pattern coding.^16^

## RESULTS

### Study Participant Profiles

#### Service Recipients

The average age of participants was 42 years, with an even mix of self-identified males and females. Regarding race, the sample was mostly homogenous with only one participant identifying as black and the remainder as white. The referral sources for these participants included counselors involved in their recovery, the court system, other social services, and friends. The participants, on average, traveled 28 miles to access KATR services; however, during the COVID-19 pandemic, most services were conducted via phone and internet. Ten out of the 12 participants noted that they had access to internet.

#### Program Coordinators

Three program coordinators of KATR also provided their insights. Each of the coordinators had lived in the local community their entire lives and had previously worked in public service positions prior to employment with the agency delivering KATR.

#### Vendors

Four vendors were interviewed as part of the study. All four businesses had been in existence for several years (i.e., 10+ years). Two vendors were automotive repair garages and two were clothing stores. The business representatives noted that they were well known in the community.

### Service Recipient Impact

Participants were overwhelmingly positive about the effect that KATR has had on the lives of service recipients. Several themes emerged related to individual-level impacts of the recovery support services, including behavior change, physical and mental health improvements, relationship repair, regaining custody of children, provision of clothing, transportation, childcare, recovery support, and ability to gain employment and improve finances. These themes are detailed below.

#### Behavior Change

More than half of the service recipients shared that being part of KATR led to dramatic behavior changes and has helped them sustain the new habits learned as part of recovery from opioid addiction. For these individuals, being part of the program has provided a “fresh start,” hope for a new life ahead, and motivation to keep pushing through hardships. Service User 1 stated that “they give addicts like myself [*sic*] hope, and they gave us something to work towards…they push us to do better.” Similarly, Service User 10 shared, “I think they show a lot of people that I did run with, look at me now and they know, and they realize that it's possible for a person to change. It's possible to beat an addiction. You know, although it's a lifelong disease, this is going to help you.” The optimistic attitude is not only beneficial for the service user, but as noted by Coordinator 3, for the community at large: “They’re on the climb up, and they are determined, and they're excited, and they feel hopeful. . . . They’re working towards a better future for them [sic] and their families…in turn, if they do well, they actually benefit our community.” Some service recipients also disclosed very grave personal situations prior to beginning their recovery journey and encountering KATR: “I couldn’t stay out of jail. I was in trouble all the time with pills. . . . I’d be dead. Or in jail. . . . It saved my life and got my family back” (Service User 12). Each of the coordinators noted perseverance as a shared strength of their service users.

#### Health Improvements

People with substance use disorders often experience negative physical and mental health impacts. One female client (Service User 7) revealed that before entering recovery, she was “on death row” and had “given up on life.” Becoming pregnant was instrumental in her recovery and KATR helped her stay the course. Additionally, several KATR clients shared that loss of teeth is common, and KATR was able to help them with the cost of proper dentures. Not only does replacing their teeth assist with improving their physical health, but it also improves their mental health:

My absolute favorite thing is the dental. It has given them so much self-confidence. . . . I can’t even tell you how many clients that I have had that have not even had teeth in so long—years and years. And I mean, I see a picture of them smiling. . . . ‘Thank you so much. I feel great about myself.’ . . . Anyone knows once you feel good about yourself, and your self-confidence goes up, that’s when you’re able to do a lot more. (Coordinator 3)

Regarding mental health, service recipients indicated that their self-esteem, confidence, and self-image were at an all-time low once they entered recovery—and coordinators concurred. KATR has helped service recipients gain back their lost self-worth: “I think that it is a beneficial thing…it makes you feel better about yourself and, in that program, especially in a place like Southeast Kentucky, it means a lot, and it’s helpful” (Service User 3).

#### Relationship Repair

Service recipients and program coordinators indicated that behaviors and decisions associated with addiction often had detrimental impacts on the relationships between people with substance use disorders and the family and friends in their lives. In some of the cases, service recipients had lost custody of or relationships with their children due to their use of opioids and the associated outcomes (e.g., incarceration, homelessness). As a result, grandparents were raising their grandchildren. Working with KATR and other service providers (e.g., counselors), some of the service recipients indicated that they had repaired relationships with their children and/or regained custody. Service recipients also felt that their parenting skills and knowledge had improved. A few of the service recipients discussed their repaired relationships and the guilt they carried with it, such as Service User 5, who noted that KATR and addressing their substance use issues “give me new direction, give me my relationships back that . . . I really don’t even deserve to have. . . . My kids and stuff that I walked out on, . . . they give me another chance. . . . It’s unbelievable how people forgive you.”

#### Provision of Transportation Assistance

The transportation assistance provided by KATR was one of the most-cited services making an impact. The participants of this study are from rural eastern Kentucky, where two-lane roads, hills, mountains, and many miles in between small towns are common. The service recipients were traveling anywhere from 3 to 60 miles to access KATR—and likely other providers and basic needs. Thus, with minimal public transportation options, personal transportation is a necessity for most people. However, in the case of many service recipients, their cars were older and/or in need of costly repairs; without disposable income, they cannot fix them, resulting in negative consequences. The service recipients recognized the importance of a vehicle to their recovery and were grateful to KATR and the vendors providing repair services.

#### Provision of Clothing

Despite being a basic need, clothing was too expensive for several KATR clients: “I have three kids. . . . I gotta do without clothes, you know, because my kids come first” (Service User 7). This lack of proper clothing may have prevented some service recipients’ recovery, as noted by coordinators and vendors alike:

We do employment clothing for people that have jobs or that just recently get a job. I recently issued boots. That was quite expensive. The gentleman could not get the boots, and he’s like, ‘That helped me keep my job. I was able to start that job, and I was able to keep it.’ (Coordinator 3)

Having decent and proper clothing is part of maintaining one’s dignity and worth in society, and the experiences of some service recipients obtaining the clothing vouchers signifies how much they have lost to addiction and the gratitude to regain self-pride:

He was like, ‘All I wanted was to have a job, and I want it to see my son.’... I mean he was so appreciative of everything. . . . I sent him to Goodwill for clothing. Most people would be like, ‘Really? Goodwill?’, but he was like, ‘Thank you so much. I’m so excited to go and get me some pants.’ (Coordinator 2)

#### Provision of Childcare

A host of community barriers impact individuals recovering from a substance use disorder in rural areas; but childcare options, particularly for those who are single parents, present a significant struggle. There are a limited number of childcare providers in Eastern Kentucky, and even when one is located, the cost can be overwhelming to a single person not making a living wage. KATR was able to provide vouchers for childcare to parents who needed them:

If you have a mom who got custody back of her child, she’s trying to get back up on her feet, she’s working, she’s not going to make enough money to pay for day care. I mean, it’s very difficult in this area, and then, too, if these women are trying to move past their family lives, they don’t want to go back to that life again with their family. They’re trying to move forward. . . . [KATR] gives them the option as well because they’re working. They’re able to pay their rent. We’re providing childcare. (Coordinator 3)

#### Provision of Recovery Support

The recovery journey is difficult and requires an immense amount of support. Service recipients noted that they face stressors of all kinds—triggers and cravings, self-guilt, and past traumas—as they strive to stay sober. While some KATR clients noted that their family and friends provide emotional and physical care, others are not able to utilize those relationships. Some service recipients have lost loved ones to overdoses, while others must distance themselves from people who continue to use substances, as removing negative influences is critical to recovery. For some families in rural Eastern Kentucky, substance use is generational, and leaving behind one’s family is distressing. KATR coordinators fill that void of a support system––and give extra support to others who still have family and friends: “It has helped folks and kind of sent them on a new path. Gave them encouragement to go forward into a beautiful place” (Vendor 3).

#### Ability to Gain Employment and Improve Finances

The rural KATR site is in a persistently poor county. As such, financial vulnerability is a major challenge. Stresses of a financial nature can wreak havoc in the lives of vulnerable populations, especially those with a substance use disorder. Many KATR clients are starting over in most aspects of their lives, as opioid addiction cost them in many ways—including the loss of employment and assets. As discussed, KATR provides vouchers for clothing, childcare, healthcare costs, and transportation, among other needs. Several participants (service recipients, coordinators, and vendors alike) shared that the cost of buying a new tire, required work shoes, or dentures could be detrimental to individuals on the road to recovery amidst persistent poverty. Many people have options to deal with life’s inevitable problems, but people in poverty often do not: “I’ve had them sit in front of us and cry . . . because we’ve helped them. . . . I guess we take a lot of things for granted. . . . I’ve got a credit card . . . but they don’t. . . . They don’t have the cash, or they gotta pay for something else and do without” (Coordinator 1). The support through vouchers has created some financial stability for the service recipients.

One noted that the vouchers “gave me a little extra start, gave me that extra income…that I would have had to either take a loan or I would have had to borrow...kinda gave me that foot forward” (Service User 9). Further, the support from KATR—financial and emotional—provided numerous service recipients the ability and confidence to obtain employment:

I think it works great. I think it’s helped me get back on track. It really has . . . I landed this job. I’ve been with it for the last year and a half ... in the past I never did care. I never did really worry about to get promoted and things. . . . I probably I shouldn’t be here, but, you know, it’s just really taught me to take care of business and how to manage my life better. (Service User 10)

## DISCUSSION

Residents in Appalachian areas are face economic decline, persistent and extreme poverty, and lack of access to health care and prevention, all of which can contribute to hardship for those living with an opioid use disorder and seeking recovery. Kentucky has the ninth-highest rate of opioid-involved overdoses in the U.S., and the county where KATR was implemented has seen a rate of age-adjusted overdose-related deaths that is 120% higher than the national average as recently as 2018.^17^–^18^ It is vital that recovery programs provide support that not only improves the lives of the individual but also enhances the community overall.

Coordinators indicated that this challenge could be due to lack of motivation, homelessness, no means to connect (e.g., no phone/internet), or other family challenges. KATR meets the basic needs of their participants and the surrounding community, but it ultimately goes beyond this to fill gaps in the community. These gaps are felt not only by those receiving services, but also by those providing services. Many participants remarked that without KATR, they would not have received care for physical or mental health needs, such as dental services. They would also be without additional support, such as clothing needed for activities like job interviewing. With these services provided to them, service users experienced improved health, self-esteem and self-worth, as indicated in the interviews. The changes to self-esteem are felt not only by participants, but also by the coordinators and vendors who work with them.

Many participants reported that the services provided by KATR helped them regain custody and improve their parenting skills. Alongside this, KATR provides childcare so parents can seek regular employment and pay their rent, without having to worry about the added exponential cost of childcare. Participants enrolled in KATR reported seeing their own strength, and ultimately having that strength is noticed by both KATR staff and vendors.

In turn, the larger community feels the impact of KATR. This program is available in an area that has been ravaged by the opioid epidemic; it is therefore essential that it addresses stigma and helps change community attitudes toward those living with a substance use disorder. Vendors and service providers alike noted that the community has become more compassionate toward those living with opioid use disorder. The vouchers have been beneficial for the community’s small businesses and ultimately help overcome systemic problems in this Appalachian area (e.g., poverty, lack of transportation, etc.). During the COVID-19 pandemic, many vendors noted that they would have had less income without the use of KATR vouchers.

Considering systemic problems, KATR works to overcome community barriers to help individuals who are changing their way of life continue to meet their recovery goals, all while addressing community needs and fostering social change. In taking a holistic approach and seeing participants as both impacting and being impacted by their communities, KATR works to bridge gaps that exist when a person desires recovery and wants to meet essential recovery goals. Funding for the program comes through the Substance Abuse and Mental Health Services Administration (SAMHSA) and is administered through the state.

It is clear that the KATR program has made substantial efforts to reduce longstanding health problems in rural counties across the state. The concentrated health disparities in this region elucidate the necessity for critical health, economic, and social intervention. In sum, KATR strives to eliminate health disparities surrounding the opioid epidemic and to provide supplemental tools that assist participants on their lifelong journey to recovery. The opioid epidemic is widespread and complex; thus, multifaceted programs and interventions such as KATR are deserving of implementation to assist in the treatment of opioid misuse and addiction. As programs are needed to assist with opioid misuse and addiction, evaluation of these programs is equally important to assess for the effectiveness and benefit to the community and individuals in which they serve.

While the KATR program has overall shown to be helpful and impactful, it is not without challenges, such as programmatic limitations, community limitations and a need for increased marketing, and providers should work to address these as the program continues.

### Limitations

Given that this study utilized qualitative research methods and evaluated a program in a specific region of Eastern Kentucky, results should not be assumed to be generalizable to other geographical areas and with other populations. While random selection was utilized to select service recipients to be sampled for the study, the research was conducted with a small sample of individuals, and participants may not be entirely representative of the service recipients, coordinators, and vendors. It is worth noting that the small sample size could be comprised of individuals who have more favorable views of the program. However, the authors were not affiliated with the KATR program and were identified as university researchers. By identifying as third-party evaluators, the research team is hopeful that participants felt autonomous enough to provide critical feedback of the program and their experiences.

## IMPLICATIONS

The purpose of this study was to understand the impact of KATR on service recipients and the barriers they encounter in their recovery journey while part of the program. Key findings from the study show that the KATR program assisted service users with provision of basic needs (clothing, health care, transportation etc.). Through the provision of these basic needs, service users indicated indirect impacts on their overall wellbeing, such as increased self-esteem and confidence, which enhanced their performance in job interviews. While there were many positive impacts, barriers still existed, such as individuals reporting guilt, stress, trauma experiences, and trying to overcome generational drug use and personal cravings. Programs are needed to help meet these needs and to reduce barriers to accessing recovery in rural areas like the area of Eastern Kentucky studied here. KATR demonstrates a useful strategy of increasing access to health-related services in predominantly rural areas through the use of social support schemes and vouchers.

**SUMMARY BOX**

**What is already known about this topic?**

The misuse of illicit and prescription opioids has become a nationwide epidemic, with particularly devastating effects in Appalachia. Many programs have arisen to address the complex determinants of addition and aid in recovery.

**What is added by this report?**

This study provides a comprehensive program evaluation of Kentucky Access To Recovery (KATR), an Eastern Kentucky-based program to provide supplemental supports to individuals in recovery from opioid use disorder. It details the experiences of service users, programmatic staff, and vendors contracted to provide services to program participants. Their interview responses—and the themes emerging from them—show that the KATR program has made substantial efforts to reduce longstanding health problems in rural counties across Kentucky.

**What are the implications for future research?**

KATR demonstrates a useful strategy of increasing access to health-related services in predominantly rural areas through the use of support schemes and vouchers. Because of the complex nature of the opioid epidemic, it is important that policymakers and health officials pursue multifaceted programs, like KATR, that combine health, economic, and social intervention. Future research should assess these programs not just in terms of individual impact, but also in respect to their impact on communities at large.

## Supplementary Information


