# Supplementary material for: Over the Hills and Through the Hollers: How One Program is Assisting Residents of Appalachian with Opioid Use Recovery
Source: J Appalach Health. 2023 Jan 1;4(3):71–86. doi: 10.13023/jah.0403.05 (PMC10655734; doi:10.13023/jah.0403.05)
Supplement: Supplementary file 1 [file 4.3.5_Jones_Additional_Files.pdf]

Appendix A – Interview Guide

Program Coordinators

Role

1. Please tell me a little about your role with KATR?
2. How long have you been in this position?
3. What are the challenges you face in your role?
4. How has COVID-19 impacted the people you serve and your role?

Service User Impact Assessment

5. What are the strengths of the service recipients?
6. How has the KATR program impacted the lives of service users?
    a. To what extent has KATR contributed positively to the lives of service users?
7. What are the biggest obstacles service users experience in their recovery?
8. What traumas have the people you serve encountered in their lives?
9. Are service users able to identify the changes that have occurred as a result of KATR?

Organizational Impact Assessment

10. To what extent do you believe KATR or programs like it are needed in Letcher and the surrounding counties?
11. Were there any changes that happened because of the KATR program that you did not expect?
    b. Have there been any unintended consequences (positive or negative) from KATR?
12. To what extent do you think the KATR program has contributed economically to Letcher and the surrounding Counties?
13. What are the strengths of KATR?
14. What are the challenges of KATR?
15. What would the community look like without KATR?
16. To what extent, if any, has the KATR program shifted attitudes or beliefs in your community regarding people with opioid use disorder or those in recovery?
17. To what extent has KATR enhanced the lives of vulnerable populations such as women and children?

Community level Assessment

18. What services are missing from Letcher and surrounding counties that are needed for people with opioid use disorder and those in recovery?
19. What is your sense of community level support for the KATR program?
20. What are the policy-level barriers that impact your role and the people you provide services to?

Vendors

Role

1. Please tell me a little about your role and how you became in contact with KATR?
2. In what ways is your organization affected by community serving organizations such as Fahe? (Or, in what ways is your position affected by KATR?)
3. How has COVID-19 impacted your community?

Impact Assessment

4. To what extent are you familiar with the Kentucky Access to Recovery program?
    a. Include description of KATR if they aren't familiar
5. To what extent do you see a need for the KATR program or programs like it in your community?
6. What changes have occurred in Letcher and surrounding counties as a result of KATR?
    b. Were there any changes that happened because of the KATR program that you did not expect?
7. To what extent do you think the KATR program has contributed economically to Letcher and the surrounding counties?
8. What are the strengths of KATR?
9. What are the challenges of KATR?
10. What would the community look like without KATR?
11. Where would service users find services if KATR did not exist?
12. Since working with KATR, have your attitudes or beliefs about people who use substances or have addictions changed in anyway?
    c. If yes, what was the point where your attitude change? Is there a moment or story that stands out?
13. Anything else you would like to share about the impact of KATR?

Service Recipients

Role

1. Please tell me a little about yourself and how you became in contact with KATR?
2. What county do you live in? (Zip code?)
3. What do you do for work?
4. Have you or one of your family members ever worked in a coal mine?
5. Do you have reliable access to internet?
6. How has COVID-19 impacted your life?
7. How has COVID-19 affected your ability to access services?

Personal Impact Assessment

8. How has the KATR program impacted your life?
    a. What changes have occurred in your life as a result of KATR?
    b. Probing questions: How did the support provided to you by the KATR program impact your relationships with family or friends?
    c. How did the support provided to you by KATR impact your financial well-being or housing situation?
9. How far did you have to travel to access KATR?
10. How did KATR support you?

11. If KATR didn't exist, would there be other resources you could access to help?
12. What have been some of the biggest barriers you've encountered in your recovery?

Community Impact Assessment

13. To what extent do you think the KATR program has contributed economically to Letcher and the surrounding counties?
14. What are the strengths of KATR?
15. What are the challenges of KATR?
16. What would the community look like without KATR?
17. To what extent, if any, has the KATR program shifted attitudes or beliefs in your community regarding people with opioid use disorder or those in recovery?
18. Anything else you would like to share about the impact of KATR?

Thank you for your time today, I appreciate your thoughts and hearing about your experience with KATR. Before we finish, may I ask you a few questions about yourself?

- Age?
- Gender?
- Race?
- Ethnicity?
- Parent or not?
- Marital Status?
- Education?
